# Evaluation of fracture resistance of class V premolar restorations reinforced with Ribbond and InFibra fibers using two placement techniques: an in vitro study

**DOI:** 10.1186/s12903-026-09423-y

**Published:** 2026-07-30

**Authors:** Possy Moustafa Abd El Aziz, Ahmed Abdelsattar Metwaly

**Affiliations:** 1 Conservative Dentistry Department, Faculty of Dentistry, Cairo University and Egyptian Russian University, Cairo, Egypt; 2 Conservative Dentistry Department, Faculty of Dentistry, Egyptian Russian University, Cairo, Egypt

**Keywords:** Fiber-reinforced composite, Fracture resistance, Polyethylene fibers, Class V cavities

## Abstract

**Aim:**

The study aim was to evaluate the fracture resistance of premolar teeth with class V cavities restored with two types of polyethylene fibers using two different techniques of fiber placement in comparison to traditional composite restoration.

**Method:**

Seventy-five intact maxillary premolar teeth were randomly divided into five groups (*n* = 15). Group 1 (control): class V cavities were filled with flowable resin composite, Beautifil flow plus F03; Group2 (Ribbond MD): Ribbond fiber placed horizontally in a mesiodistal orientation on the axial wall of the cavity; Group 3 (Ribbond OG): Ribbond fiber placed on the axial wall of the cavity in an occlusogingival orientation; Group 4 (InFibra MD): InFibra fiber placed horizontally in a mesiodistal orientation on the axial wall of the cavity; Group 5 (InFibra OG): InFibra fiber placed on the axial wall of the cavity in an occlusogingival orientation. After fiber placement, all cavities were filled with the same flowable resin composite, Beautifil flow plus F03 and cured for 10 s. Fracture resistance was assessed using a universal testing machine. Failure mode was also analyzed using magnification loupes.

**Results:**

A one-way ANOVA that was performed to assess fracture resistance across the five groups, showed no statistically meaningful differences among groups (F = 0.3034, *p* = 0.8722), with an extremely small effect size (η² = 0.0572). Post hoc analyses with Tukey’s HSD revealed that no pairwise differences were significant (*p* > 0.05). Failure mode results revealed 60% favorable fractures in the control group, Ribbond MD and Ribbond OG each demonstrated 20% favorable fractures, whereas InFibra MD and InFibra OG recorded no favorable fractures.

**Conclusion:**

The incorporation of polyethylene fibers (Ribbond or InFibra) into moderately deep Class V cavities did not significantly enhance the fracture resistance of the teeth. Furthermore, the addition of these fibers was associated with a higher frequency of unfavorable fractures.

## Introduction

Resin composites have gained significant popularity in restorative dentistry due to their superior aesthetics, durable bonding and their biocompatibility. Despite their popularity, resin composites suffer from brittleness with limited fracture resistance, which affect its capability to absorb stresses, resulting in crack propagation. Polymerization shrinkage is another flaw of composite restorations which induces internal stresses inside the restoration, causing microcracks and finally failure of restoration [1]. Premolars, especially the maxillary ones when subjected to a lateral force that applied to the cusp, the tooth acts like a lever and the cervical region acts as the fulcrum causing flexural stresses at the tooth cervix, which predispose to failure of composite restorations in class V cavities [2]. Because of the particular difficulties associated with cervical cavities in premolars, standard resin procedures may not yield consistent or durable results for cervical restorations [3]. The fiber-reinforced composite restoration was also introduced to increase durability, flexural strength, and fracture resistance of the composite restoration, and increase resin stiffness. It decreased polymerization shrinkage and provided better force distribution along the fibers, thereby improving the physical and mechanical properties of the composites [4]. Incorporating polyethylene fibers into composite restorations may provide better results when the fibers are adapted to the inner contours of the remaining tooth substrate, yielding an enhancement of the fracture protection mechanism. Polyethylene fibers also do not absorb water and are resistant to chemicals [5].

The dental field is really interested in two kinds of polyethylene fibers, Ribbond and InFibra. These fibers can make restorations better and last longer [6]. Selecting these two specific fibers allows for a comparative analysis of their distinct macro-architectures. Ribbond^®^ consists of pre-impregnated, plasma-treated ultra-high-molecular-weight polyethylene (UHMWPE) fibers with a unique leno-woven design. This weave allows forces to dissipate throughout the network without transferring stress loads back into the composite, essentially providing multiple loading paths that distribute occlusal and shrinkage stresses over a broad surface [7]. Conversely, InFibra^®^ Ribbon utilizes an interlaced or woven macro-architecture designed to improve handling and multidirectional stress distribution.

Ultimately, however, the fundamental reinforcement mechanism in both systems relies on the internally aligned crystalline microfibrils within the UHMWPE fibers [8]. The mechanical efficacy of FRCs is dictated by several critical factors, including surface treatment, fiber density, and fiber orientation relative to occlusal stresses [9]. While the general advantages of fiber reinforcement are widely acknowledged, clinical opinions regarding optimal fiber orientation and placement techniques remain highly diverse [10]. Comparing mesiodistal versus occlusogingival orientations is clinically relevant, as establishing a standardized placement protocol is necessary to maximize the mechanical performance of fiber-reinforced cervical restorations. Therefore, the aim of this study was to evaluate the fracture resistance and failure modes of premolar teeth with class V cavities restored with two types of polyethylene fibers (Ribbond and InFibra) using two different placement techniques (mesiodistal and occlusogingival) compared to traditional composite restorations. The null hypothesis stated that neither the type of fiber nor the placement technique would significantly affect the fracture resistance or the failure mode of premolar teeth with class V cavities compared to traditional composite restoration.

## Methods

Ethical approval was granted by the Ethical Committee of Faculty of Dentistry, Egyptian Russian University (No. 29) in view of the in-vitro nature of the study. Seventy-five intact caries-free premolar teeth were gathered from the ERU outpatient clinics after being extracted for orthodontic or periodontal purposes. To ensure standardization and consistent defect proportions among specimens, the maximum buccolingual dimension, mesiodistal width, intercuspal length, and occlusogingival height of each tooth were measured using a digital caliper (Tooleye, Frankfurt, Germany). Teeth of comparable size were selected, with an average buccolingual width of 10 mm, mesiodistal width of 7 mm, intercuspal distance of 6 mm, and occlusogingival height of 9 mm. To ensure consistency between and across groups, the teeth measures were standardized to ensure that they deviated from the group mean by no more than 10% [10]. The cleaned extracted teeth were disinfected in 0.5% chloramine T for 1 day, stored in distilled water at 4 °C, and embedded in self-cured acrylic resin (Acrostone TM, Cairo, Egypt) to a distance of 2 mm from the cementoenamel junction (CEJ) [11]. Using a randomization list generated by www.random.org, the selected teeth were allocated to one of the five groups (n = 15).

### Sample size calculation

A power analysis was conducted to determine the appropriate sample size for this in vitro study evaluating and comparing the fracture resistance of polyethylene fiber-reinforced composites in class V premolar cavities using two fiber types (Ribbond and InFibra) and two placement techniques, in addition to a control group without fibers. Based on previously published data [12], Ribbond fiber-reinforced restorations demonstrated higher fracture resistance (mean = 1075.6 N) compared to fiber-reinforced restorations with Interlig (mean = 881.6 N), with an effect size (Cohen’s d) of 1.83, indicating a large expected difference. Considering the five-group design, effect-size adjustment for ANOVA (Cohen’s f ≈ 0.456), a significance level (α) = 0.05, and a power (1–β) = 0.80, the calculated sample size was 13 teeth per group. To accommodate possible specimen loss due to handling, preparation errors, or testing anomalies, the sample size was increased to 15 teeth per group, resulting in a total of 75 extracted human premolars. Sample size calculation was performed using G^*^ Power version 3.1.9.7.

### Cavity preparation

**Fig. 1:**
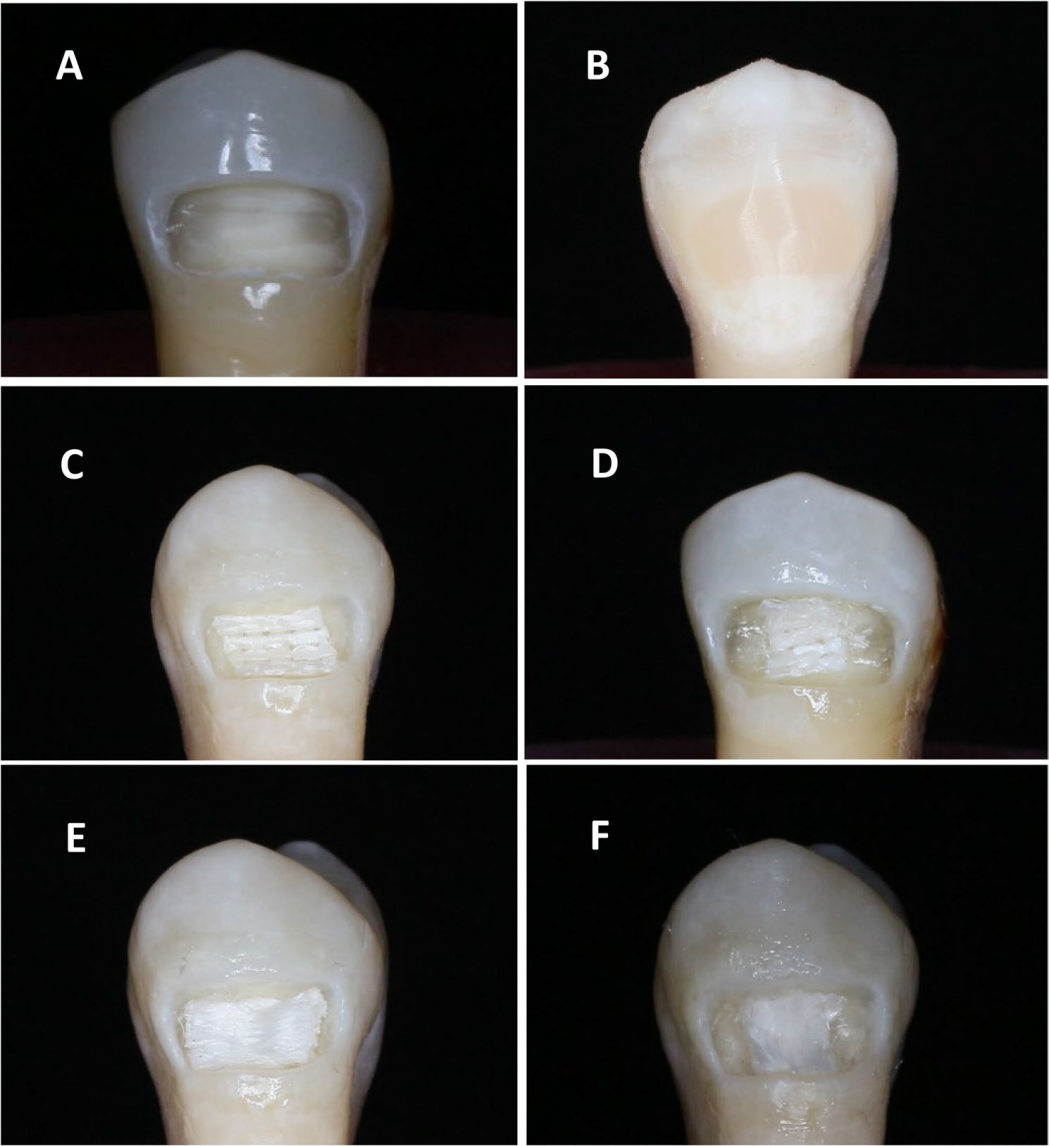
(**A**) Class V prepared cavity; (**B**) the cavity was filled by flowable resin composite only; (**C**) Ribbond fiber placed horizontally on the cavity axial wall in a mesiodistal direction; (**D**) Ribbond fiber placed vertically on the cavity axial wall in an occlusogingival direction; (**E**) InFibra fiber placed horizontally on the cavity axial wall in a mesiodistal direction; (**F**) InFibra fiber placed vertically on the cavity axial wall in an occlusogingival direction

Standardized class V cavities were prepared by one operator in the cervical region of the buccal surfaces of the selected teeth with dimensions 1.5 × 4 × 2 mm (depth, mesiodistal, occlusocervical, respectively), using an 856 round end tapered bur (Komet Italia SRL, Italy) attached to a high-speed handpiece with a suitable water cooling. The cavity dimensions were measured using a graduated periodontal probe (UNC-15, Chicago, USA). The gingival cavity margin was 1 mm above the cementoenamel junction [13]. After cleaning, the cavities were air dried (Fig. 1A).

### Restorative procedure

In accordance with the manufacturer’s instructions, each group’s enamel was selectively etched for 15 s using a 37% phosphoric acid etching gel (Scotchbond, 3 M ESPE, USA), followed by a 15 s rinsing and gently air-dried. A universal adhesive (BeautiBond Xtreme, Shofu Inc., Japan) was applied using a disposable microbrush. A light curing unit (LED.F, Woodpecker, China) with an intensity of 1600–1800 mW/cm2 was used to cure the surface for 10 s after it had been air dried for three seconds with mild airflow and then with stronger airflow until the surface appeared glossy with no detectable adhesive movement.

Group 1 (Control): The cavity in the control group was filled with Beautifil flow plus F03 (Shofu Inc., Japan), a flowable resin composite, and allowed to cure for 10 s (Fig. 1B) [14]. Group 2 (Ribbond MD): The axial wall was covered with a layer of flowable composite. A leno waved ultra-high-molecular-weight polyethylene ribbon fiber (Ribbond-Ultra; Ribbond Inc., Seattle, WA, USA) was taken out from the container using cotton pliers. After determining the necessary length using tin foil in compliance with the manufacturer’s instructions, specialized scissors provided in the Ribbond package was used to cut the fiber piece to guarantee a precise cut. Before the fiber was placed in the cavity, it was moistened with an unfilled resin (Ribbond wetting resin, Ribbond Inc., Seattle, WA, USA). The extra resin was forced out of the fiber using a plastic tool that was positioned in line with the length of the fiber [15]. The cut Ribbond fiber was positioned horizontally in a mesiodistal orientation on the axial wall of the cavity. Using a plastic tool, the fiber was carefully adjusted to fit the wall, making sure it did not reach the cavity margins, then light curing was done for 10 s. The same flowable resin composite was used to fill the remaining cavity, and it was cured for 10 s (Fig. 1C).

Group 3 (Ribbond OG): adhered to the identical protocol for tooth restoration as group 2, with the exception that the fiber was positioned on the axial walls in an occlusogingival orientation (Fig. 1D). Group 4 (InFibra MD): adhered to the same protocol for tooth restoration as group 2, but a different kind of polyethylene fiber was used. Following the manufacturer’s guidelines, a bioloren weave polyethylene fiber (InFibra; Bioloren Inc., Volta, Italy) was trimmed and moistened with universal adhesive (BeautiBond Xtreme, Shofu Inc, Japan). Subsequently, positioned in the cavity on the axial wall in the mesiodistal orientation as demonstrated in group 2 (Fig. 1E). Group 5 (InFibra MD): adhered to the same protocol for tooth restoration as group 4, but InFibra fibers were placed on the axial wall of the cavity in an occlusogingival direction (Fig. 1F). Restorations were finished using fine grit tapered diamond stones (Microdont, Brazil), then polishing was done by rubber points and bristle brushes with ultrafine Microdont polishing paste (Microdont, Brazil). Polishing was performed at low-speed under copious water cooling to achieve a highly polished surface [16]. All materials used in the study are listed in (Table 1).

**Table 1:** Specifications, composition, manufacturer, and lot number of the material

Specification	Material	Composition	Lot number	Manufacturer
BeautiBond Xtreme	Universal dental adhesive system	Acetone, Silane coupling agent, Bis-GMA, acid monomer, distilled water, and TEGDMA	IFU-000278	Shofu Inc, Kyoto, Japan
Wetting resin	Ceramage “modelling liquid”	Dimethyl Aminoethyl Methacrylate, UDMA and Others	112,425	Shofu Inc, Kyoto, Japan
Polyethylene fibers	Ribbond	Leno weaved plasma-treated high-molecular-weight polyethylene fibers	9002-88-4	Ribbond ULTRA, Ribbond Inc., USA
Polyethylene fibers	InFibra	Bioloren weaved plasma-treated high-molecular-weight polyethylene fibers	12/01	Bioloren Inc., Volta, Italy
Beautifil flow plus F03	Fluoride Releasing flowable resin composite	Fluoroboroaluminosilicate glass-based Bis-GMA, TEGDMA, S-PRG filler, polymerization initiator, and pigments	032434	Shofu Inc, Kyoto, Japan

### Fracture resistance examination

A universal testing apparatus (Instron 3343, Norwood, MA, USA) was utilized. The specimens mounted were exposed to an axial compressive force with a 4 mm steel ball [17–19]. A crosshead velocity of 1 mm per minute was applied. The steel ball was held above the center of the tooth, parallel to its long axis, until it touched both the buccal and lingual cusp slopes and the occlusal surface simultaneously.

The use of axial compressive loading was deliberately selected as a standardized baseline method to directly evaluate the ultimate static fracture threshold of the restorative unit [11, 20, 21]. A force versus extension curve was generated for each specimen following the application of a load until fracture took place. The fracture threshold, measured in Newtons (N), was established as the initial force that resulted in the fracture [22, 23].

### Analysis of failure mode

Using magnification loupes (3.5×), fractured samples were examined to look into failure mode. Fractures were deemed (unfavorable) when the fracture line extended greater than 1 mm apical to the cemento-enamel junction, and (favorable) if the fracture line was situated above the cemento-enamel junction [24].

### Analysis of statistics

MedCalc Statistical Software version 22 (MedCalc Software Ltd, Ostend, Belgium) was used to examine the data. The fracture resistance data’s normality was assessed using the Shapiro-Wilk test, which revealed a normal distribution (*p* > 0.05). The data for fracture resistance were shown as mean ± standard deviation (SD). One-way ANOVA was used to examine fracture resistance between groups, and Tukey’s post hoc test was used for pairwise comparisons. The effect size was determined as η² for ANOVA. Frequencies and percentages [n (%)] were used to represent categorical data (failure mode). Group associations were evaluated using the chi-square test of independence, and effect size was computed using Cramer’s V. Spearman’s rank correlation evaluated the relationship between fracture resistance and failure mode in each group. Every test was two-tailed, and statistical significance was set at *p* < 0.05 [25].

## Results

### Resistance to fracture (N)

**Fig. 2:**
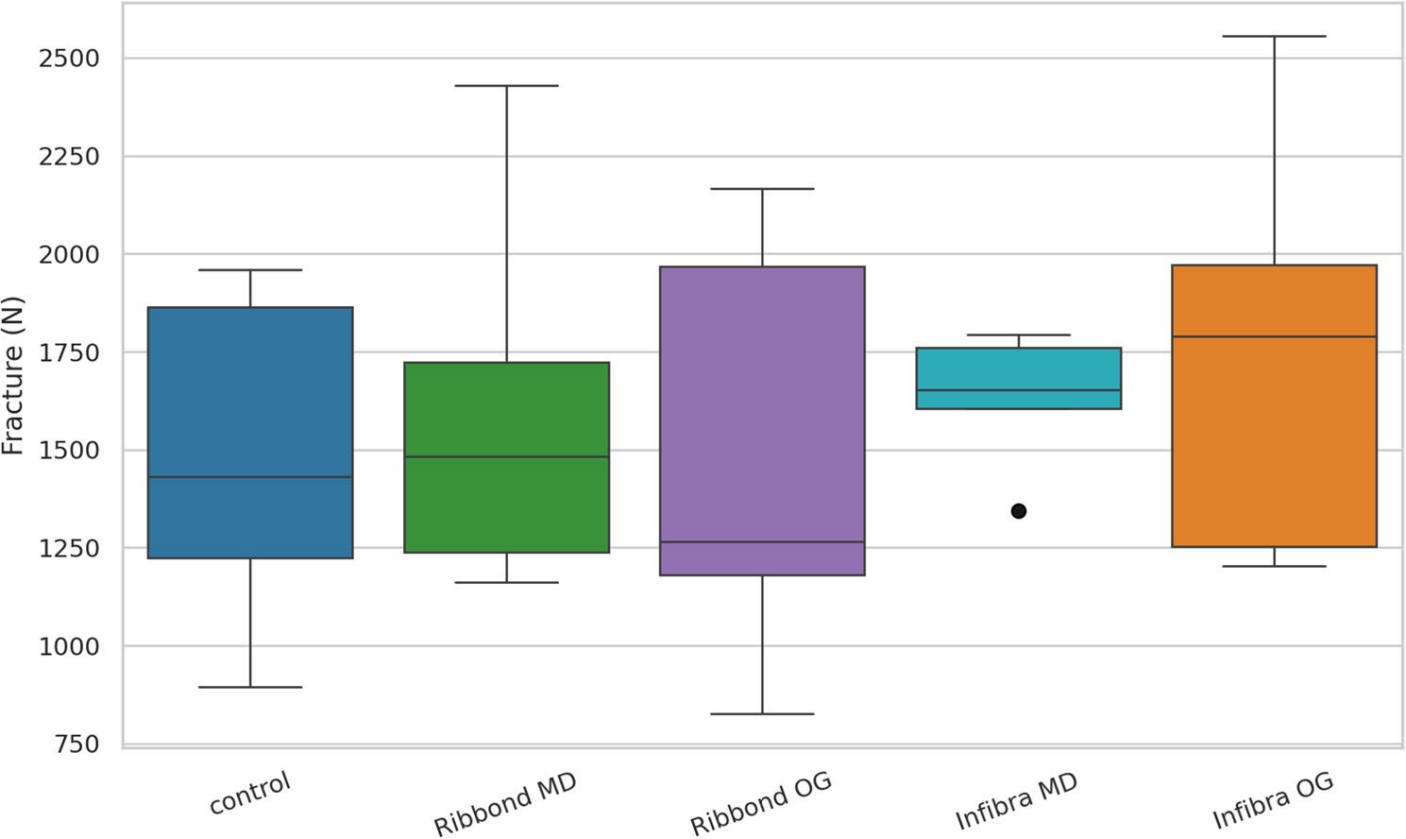
Box plot of fracture resistance among groups

Average fracture resistance values (in N) were: Control (1474.4 ± 443.6), Ribbond MD (1607.6 ± 509.9), Ribbond OG (1481.2 ± 564.0), InFibra MD (1630.8 ± 178.1), and InFibra OG (1754.6 ± 557.9). The five groups’ fracture resistance was evaluated using a one-way ANOVA. The examination showed no statistically meaningful differences among groups (F = 0.3034, *p* = 0.8722), with an extremely small effect size (η² = 0.0572). No pairwise differences were significant (*p* > 0.05) according to post hoc analysis using Tukey’s HSD (Table 2) (Fig. 2).

**Table 2:** The groups’ fracture resistance means, standard deviation, minimum, and maximum

Group	Mean ± SD	Min–Max
Control	1474.4 ± 443.62	894–1959
Ribbond MD	1607.6 ± 509.86	1162–2430
Ribbond OG	1481.2 ± 563.97	826–2166
InFibra MD	1630.8 ± 178.11	1343–1793
InFibra OG	1754.6 ± 557.88	1204–2555
F test	0.3034	
P value	0.8722	
Effect size (η²)	0.0572	

### Failure mode findings

**Fig. 3:**
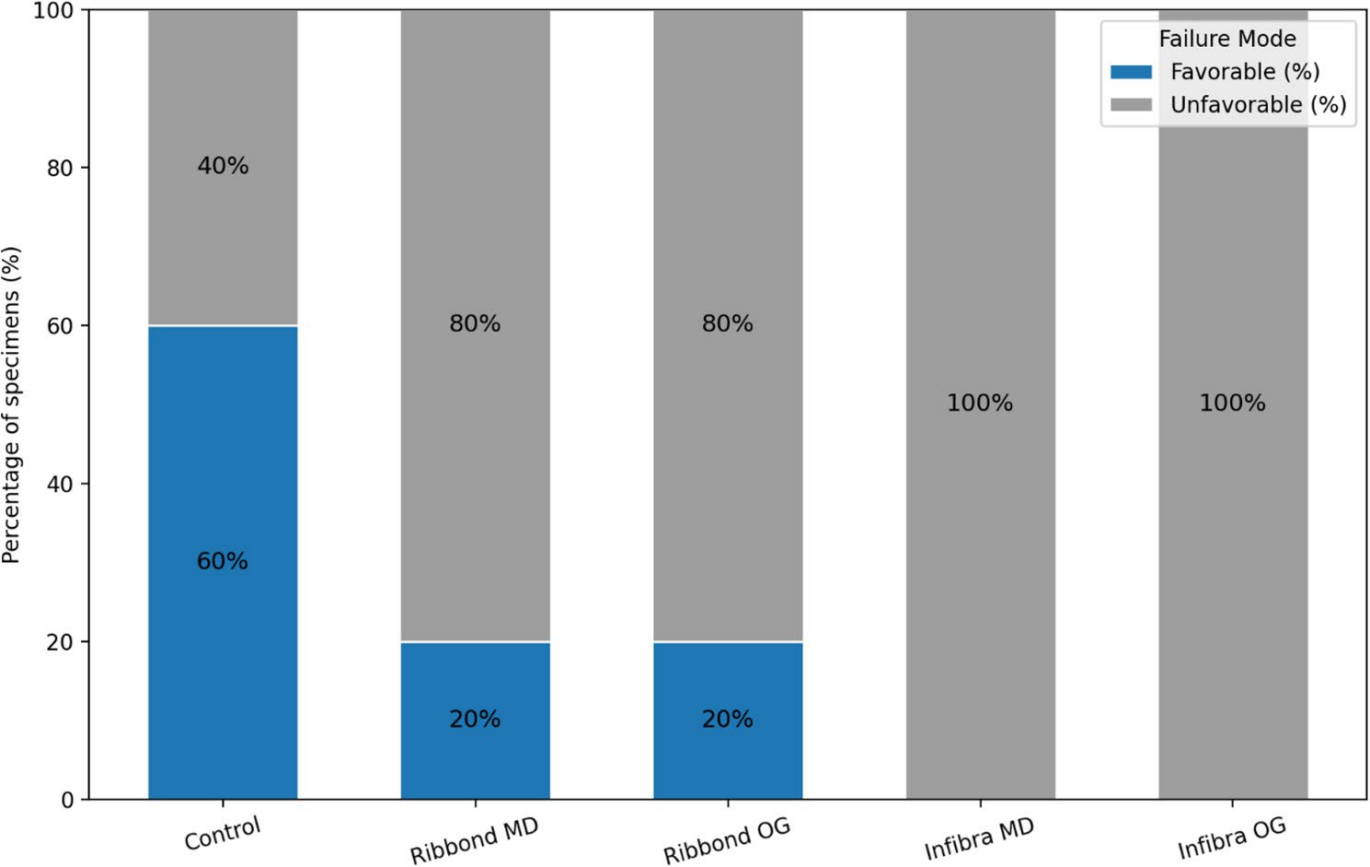
100% stacked column showing failure mode distribution among groups

**Fig. 4:**
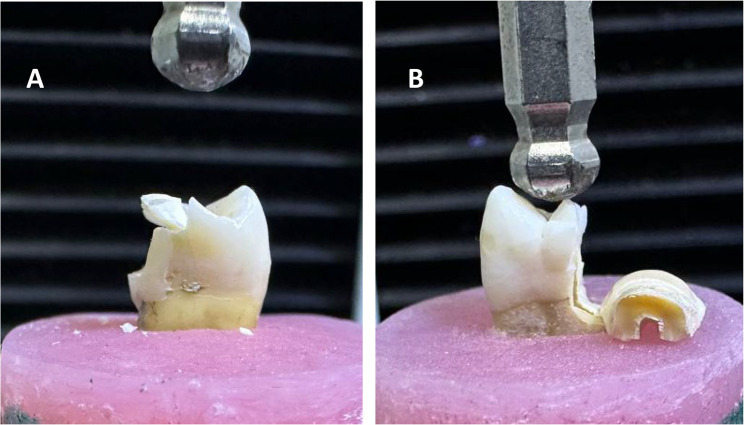
Failure mode: (**A)** Favorable; **(B)** Unfavorable

The failure mode distribution among the five groups (Control, Ribbond MD, Ribbond OG, InFibra MD, and InFibra OG) was as such: Control displayed 60.0% favorable fractures, Ribbond MD and Ribbond OG each demonstrated 20.0% favorable fractures, whereas InFibra MD and InFibra OG recorded no favorable fractures. The comprehensive chi-square analysis revealed a significant correlation between group and failure mode, (*p* = 0.0002) with a moderate-to-large effect size (Cramér’s V = 0.548) (Table 3) (Figs. 3 and 4).

**Table 3:** Frequency and percentage of failure mode among groups

Group	Favorable	Unfavorable
Control	9 (60.0%)	6 (40.0%)
Ribbond MD	3 (20.0%)	12 (80.0%)
Ribbond OG	3 (20.0%)	12 (80.0%)
InFibra MD	0 (0.0%)	15 (100.0%)
InFibra OG	0 (0.0%)	15 (100.0%)
Total	**15 (20%)**	**60 (80%)**
P value	**0.0002**	
Effect size (Cramér’s V)	**0.5477**	

### Correlation between fracture resistance and failure mode among groups

In the Control group, the relationship was weak and not statistically significant (ρ = −0.289, 95% CI: −1.000 to 0.968, *p* = 0.638). For Ribbond MD, a moderate negative correlation was noted (ρ = −0.707, 95% CI: −1.000 to − 0.707), although this did not achieve statistical significance (*p* = 0.182). Ribbond OG exhibited a slight negative correlation (ρ = −0.354, 95% CI: −1.000 to 0.395, *p* = 0.559). In both the InFibra MD and InFibra OG groups, the failure mode showed no variability (all samples were unfavorable), rendering correlation estimation unfeasible. Fracture resistance and failure mode did not generally show a significant monotonic association in any group; a negative ρ indicates that specimens with higher fracture resistance often had more undesired failures.

## Discussion

Class V cavities located on the cervical third of teeth poses several challenges for restorative dentistry, including moisture control and exposure to high mechanical and thermal stresses. Success of restoration in class V cavities mainly depends on the restorative material adaptation to the cavity walls and the restorative material ability to withstand occlusal and lateral forces without fracture. Polymerization shrinkage of composites creates contraction stresses leading to decreased fracture resistance and restoration failure [26]. Therefore, selecting an appropriate resin composite with a compatible adhesive system is important for restoration success. Fortunately, reinforcing composite restorative materials with ultra-high-molecular-weight polyethylene fibers resulted in increased strength and toughness of these types of restorations. Ribbon systems of leno weaved ultra-high-molecular-weight polyethylene fibers are developed to enhance the toughness of resin composite restorations with longer durability and greater damage tolerance. These continuous fibers are adapted in close proximity to the sound tooth structure with multiple directional and mesh-like intersecting fibers lead to redistribution of the masticatory forces over a larger area of composite restoration, altering the interfacial stresses with a higher elastic modulus and lower flexural modulus. Each fiber acts as crack stopper by changing the stress direction that consequently dissipates the strain.

Like the dentin-enamel complex, which helps dentin and enamel to work in strain harmony together [25]. Hence, in the present study, flowable composite restoration in combination with fiber reinforcement were used to gain the benefit of both better adaptation and stress distribution. Two types of polyethylene fibers were used with two different techniques of fiber placement in comparison to traditional flowable composite restoration. Ribbond fibers were impregnated with wetting resin before placement because it helps in merging of the fiber with the polymer matrix. This avoids oxygen entrapment, which interfere with resin polymerization, decreasing the amount of residual monomer and improving the bond strength. While, InFibra fibers were moistened with universal adhesive in accordance with the manufacturer’s recommendations [27].

Fracture resistance is one of the important preliminary characteristics of dental restorative materials. Fracture at the interface between the tooth structure and composite restoration of cervical lesions is mostly caused by the polymerization shrinkage of composite restoration and the difference in their moduli of elasticity [12]. The fracture resistance and failure patterns of Class V flowable composite restorations were evaluated in this research in relation to different fiber reinforcing materials (Ribbond and InFibra) and their alignment (mesiodistal and occlusogingival). Since there was no statistically significant difference seen between the groups, the fracture resistance null hypothesis was adopted. Nevertheless, the null hypothesis concerning failure mode was dismissed, as notable differences were detected, with the control group showing more advantageous fracture patterns than the fiber-reinforced groups. The lack of significant improvement in ultimate fracture resistance aligns with other studies suggesting that fiber reinforcement may not consistently provide distinct mechanical benefits depending on cavity design and dimensions [13, 17]. A critical interpretation of these findings points to several contributing factors. First, the limited dimensions of the standardized Class V cavities (1.5 mm deep) may have restricted the fibers’ ability to operate as an effective stress-absorbing mechanism.

In shallow defects where restorative volume is low, the fiber mesh occupies a substantial portion of the cavity. This limits the bulk of the overlying composite, meaning the overall stiffness of the tooth-restoration complex might not significantly surpass that of a robustly bonded modern flowable composite [23]. Furthermore, contemporary flowable materials like the Beautifil Flow Plus used here exhibit a lower modulus of elasticity and enhanced mechanical properties compared to older hybrid composites.

This allows them to adapt efficiently and flex with the tooth structure under load, potentially matching the performance of a fiber-reinforced unit in a static test [23, 24]. Perhaps the most striking finding was the distribution of failure modes. It is frequently hypothesized in the literature that fiber reinforcement acts as a “crack-stopping” mechanism that promotes favorable, repairable fractures [12, 17]. However, our experimental data revealed the opposite. The control group experienced 60% favorable fractures, whereas the Ribbond groups suffered 80% unfavorable fractures, and the InFibra groups suffered 100% unfavorable fractures. To explain these catastrophic patterns, we hypothesize that the incorporation of a highly stiff ultra-high-molecular-weight polyethylene fiber network fundamentally altered the biomechanical behavior of the tooth. As previously demonstrated in finite element analyses by Park et al. [2], Class V restorations are situated at the cervical fulcrum of the tooth, an area already prone to severe flexural stress concentration. By creating an exceptionally stiff, cohesive unit at this critical fulcrum, the fiber-reinforced restorations likely prevented the composite from acting as a sacrificial layer. Instead of failing favorably (e.g., adhesive debonding under extreme compressive stress), this highly rigid reinforced complex may have directed the immense structural stress directly into the underlying, less stiff root dentin. Once the physiological threshold of the dentin was breached, the released energy manifested as an irreparable vertical root fracture. It must be noted, however, that because stress distribution was not directly measured via finite element analysis in this study, this mechanism remains a biomechanical hypothesis intended to explain the observed in vitro fracture patterns [24, 28].

It is crucial to interpret the findings of this study within the context of its in vitro limitations, which restrict direct clinical extrapolation. First, the specimens were rigidly embedded directly into self-curing acrylic resin without simulating the periodontal ligament (PDL). As highlighted by Szabó et al. [24], load-to-fracture mechanical testing is highly sensitive to embedment materials. The natural PDL plays a critical physiological role in dampening and distributing occlusal forces along the root. The absence of an elastomeric PDL simulation in this rigid setup likely created an artificial stress concentration exactly at the cementoenamel junction, which may have exacerbated the high incidence of catastrophic vertical root fractures observed in the reinforced groups. Second, this research utilized a continuous static compressive force until failure.

In the dynamic oral environment, however, restorations predominantly fail due to cyclic masticatory fatigue and oblique excursive forces over time, rather than a single maximum traumatic load. Finally, this protocol lacked artificial aging (thermocycling), which is necessary to understand how thermal expansion and water sorption impact the long-term bond degradation of fiber-reinforced restorations. Future clinical trials and dynamic cyclic-loading in vitro models are necessary to validate these findings.

## Conclusion

Within the limitations of this in vitro study, the addition of polyethylene fibers (Ribbond or InFibra) using either mesiodistal or occlusogingival placement techniques in moderately deep Class V cavities did not significantly enhance the ultimate fracture resistance of the teeth. Furthermore, fiber reinforcement was associated with a significantly higher rate of unfavorable fracture modes.

## Data Availability

The datasets used and/or analyzed during the current study are available from the corresponding author on reasonable request.

## Abbreviations

FRC: Fiber-Reinforced Composite
UHMWPE: Ultra-High-Molecular-Weight Polyethylene
CEJ: Cementoenamel Junction
MD: Mesiodistal
OG: Occlusogingival
ANOVA: Analysis of Variance
SD: Standard Deviation
PDL: Periodontal Ligament
